# Plasma Fatty Acid Profiles Modulate PPARγ Expression in Adipose Tissue: A Lipidomic Insight Into Obesity‐Related Metabolic Dysregulation

**DOI:** 10.1002/edm2.70080

**Published:** 2025-09-18

**Authors:** Maryam Sanoie, Farshad Teymoori, Raziyeh Abooshahab, Mahdi Akbarzadeh, Golaleh Asghari, Emad Yuzbashian, Mehdi Hedayati, Alireza Khalaj, Maryam Zarkesh

**Affiliations:** ^1^ Cellular and Molecular Endocrine Research Center, Research Institute for Endocrine Molecular Biology, Research Institute for Endocrine Sciences Shahid Beheshti University of Medical Sciences Tehran Iran; ^2^ Department of Nutrition, School of Public Health Iran University of Medical Sciences Tehran Iran; ^3^ Nutrition and Endocrine Research Center Research Institute for Endocrine Sciences, Shahid Beheshti University of Medical Sciences Tehran Iran; ^4^ Curtin Medical School Curtin University Bentley Australia; ^5^ Department of Agricultural, Food and Nutritional Science University of Alberta Edmonton Alberta Canada; ^6^ Tehran Obesity Treatment Center, Department of Surgery Shahed University Tehran Iran

**Keywords:** fatty tissue, free fatty acids, lipidomic, nondiabetic adults, PPARγ

## Abstract

**Aim:**

This study aimed to investigate the relationship between plasma fatty acids (FAs), FA‐derived factors and PPARγ expression in visceral and subcutaneous adipose tissues (VAT and SAT) of obese and nonobese adults.

**Methods:**

This cross‐sectional study involved 167 adults aged 19 to 65. Samples of VAT and SAT were obtained during elective abdominal surgeries. Participants were divided into two groups: nonobese (BMI < 30 kg/m^2^) and obese (BMI ≥ 30 kg/m^2^). Anthropometric and biochemical measurements were taken, and plasma fatty acids (FAs) were analysed using gas chromatography flame ionisation detection (GC/FID). PPARγ mRNA levels were measured through real‐time RT‐qPCR.

**Results:**

Obese individuals had higher PPARγ gene expression in both VAT and SAT compared to nonobese participants (*p* < 0.001). Eighteen FFAs and three new FA‐derived factors were identified in both groups, accounting for 69% of the variance in nonobese individuals and 71% in obese individuals. After adjusting for confounding factors, saturated FA (SFA) was associated with PPARγ expression in the SAT of the nonobese group (*β* = −0.12, *p* = 0.019). Additionally, total FAs (*β* = −0.02, *p* = 0.017), SFA (*β* = −0.06, *p* = 0.048), monounsaturated FA (MUFA) (*β* = −0.08, *p* = 0.020), polyunsaturated FA (PUFA) (*β* = −0.03, *p* = 0.039) and omega‐6 FA (*β* = −0.03, *p* = 0.040) were associated with VAT PPARγ expression among obese individuals. Conversely, an inverse correlation was observed between factor I of FAs and SAT PPARγ expression in nonobese individuals (*β* = −0.15; *p* = 0.027).

**Conclusion:**

These findings suggest that alterations in plasma FA profiles are associated with PPARγ gene expression, particularly in obese individuals. This fact highlights the potential role of dietary FAs in metabolic regulation and health issues related to obesity.

## Graphical abstract

This study examined the link among plasma fatty acids (FAs), FA‐derived factors and PPARγ expression in visceral and subcutaneous adipose tissues (VAT and SAT) of obese and nonobese adults. This cross‐sectional study involved 167 adults aged 19–65. VAT and SAT samples were collected during surgeries. Participants were grouped into two categories: nonobese (BMI < 30) and obese (BMI ≥ 30). Measurements included anthropometric, biochemical, plasma FAs via GC/FID, and PPARγ mRNA via RT‐qPCR. Obese subjects exhibited higher PPARγ expression in VAT and SAT (*p* < 0.001). Eighteen FFAs and three new FA‐derived factors explained 69% of the variance in nonobese and 71% in the obese groups. Adjusted analysis showed that SFA correlated with PPARγ in SAT of nonobese individuals (*β* = −0.12, *p* = 0.019). In obese individuals, total FAs, SFA, MUFA, PUFA and omega‐6 FAs correlated inversely with VAT PPARγ. In nonobese individuals, FAs factor I was inversely related to SAT PPARγ (*β* = −0.15, *p* = 0.027). Changes in plasma FA profiles are linked to PPARγ gene expression, especially in obesity, indicating dietary FAs may affect metabolic regulation and health.

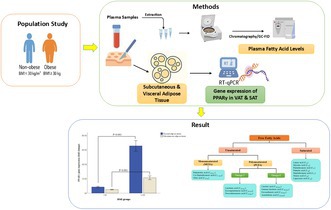

## 1 Introduction

Obesity, defined by a high body mass index (BMI), poses a serious public health challenge. It is closely linked to a greater risk of cardiovascular disease, diabetes and cancer, leading to a notable reduction in both quality of life and life expectancy [1]. In recent decades, the global prevalence of obesity has surged dramatically, reaching epidemic levels and continuing to rise at an alarming rate [2]. The development of obesity involves interactions between behavioural, environmental and genetic factors, resulting in impaired endocrine signalling in adipocytes. This dysregulation plays a crucial role in the pathophysiology of obesity‐related health complications such as diabetes and/or insulin resistance [3, 4].

One of the essential regulators of adipose tissue function is peroxisome proliferator‐activated receptor γ (PPARγ), which serves as a crucial transcriptional regulator of metabolic processes. Its activity is influenced by interactions with steroids, thyroid hormones, vitamins, metabolites, lipids and xenobiotics [5]. As a central component of the nuclear receptor superfamily, PPARγ forms heterodimers with the 9‐cis‐retinoic acid receptor (RXR). These complexes bind to specific response elements within gene promoter regions, regulating the transcription of genes essential for adipocyte differentiation and lipid metabolism [6]. PPARγ activation promotes the expression of adipocyte‐specific genes, leading to lipid droplet formation and the development of adipocyte‐like features in differentiated fibroblast cells [7]. Interestingly, studies in mice have shown that heterozygous deficiency in PPARγ leads to reduced fat mass and smaller adipocytes [8], while there was a concomitant reduction in PPARγ mRNA in the obese mice compared to its level in wild‐type mice [9]. These conflicting findings regarding PPARγ gene expression in adipose tissue highlight the need for further investigation into the role of this gene.

White adipose tissue consists of two main depots: subcutaneous adipose tissue (SAT) and visceral adipose tissue (VAT), which have distinct metabolic characteristics. VAT is more metabolically active, sensitive to lipolysis and adrenergic stimulation and has higher insulin‐stimulated glucose uptake than SAT. It contributes more significantly to insulin resistance due to its endocrine functions and proximity to the portal system, influencing systemic inflammation. Conversely, SAT is better at absorbing circulating free fatty acids (FFAs) and triglycerides and has a lower inflammatory profile, enhancing its metabolic protective effects [10, 11]. These distinct differences in metabolic behaviour, inflammatory status and PPARγ regulation motivated our inclusion of both VAT and SAT in the study. By comparing lipidomic profiles and PPARγ expression across these depots, our investigation provides a comprehensive understanding of how plasma FAs differentially modulate adipose tissue biology and contribute to obesity‐related metabolic risk.

**FIGURE 1:**
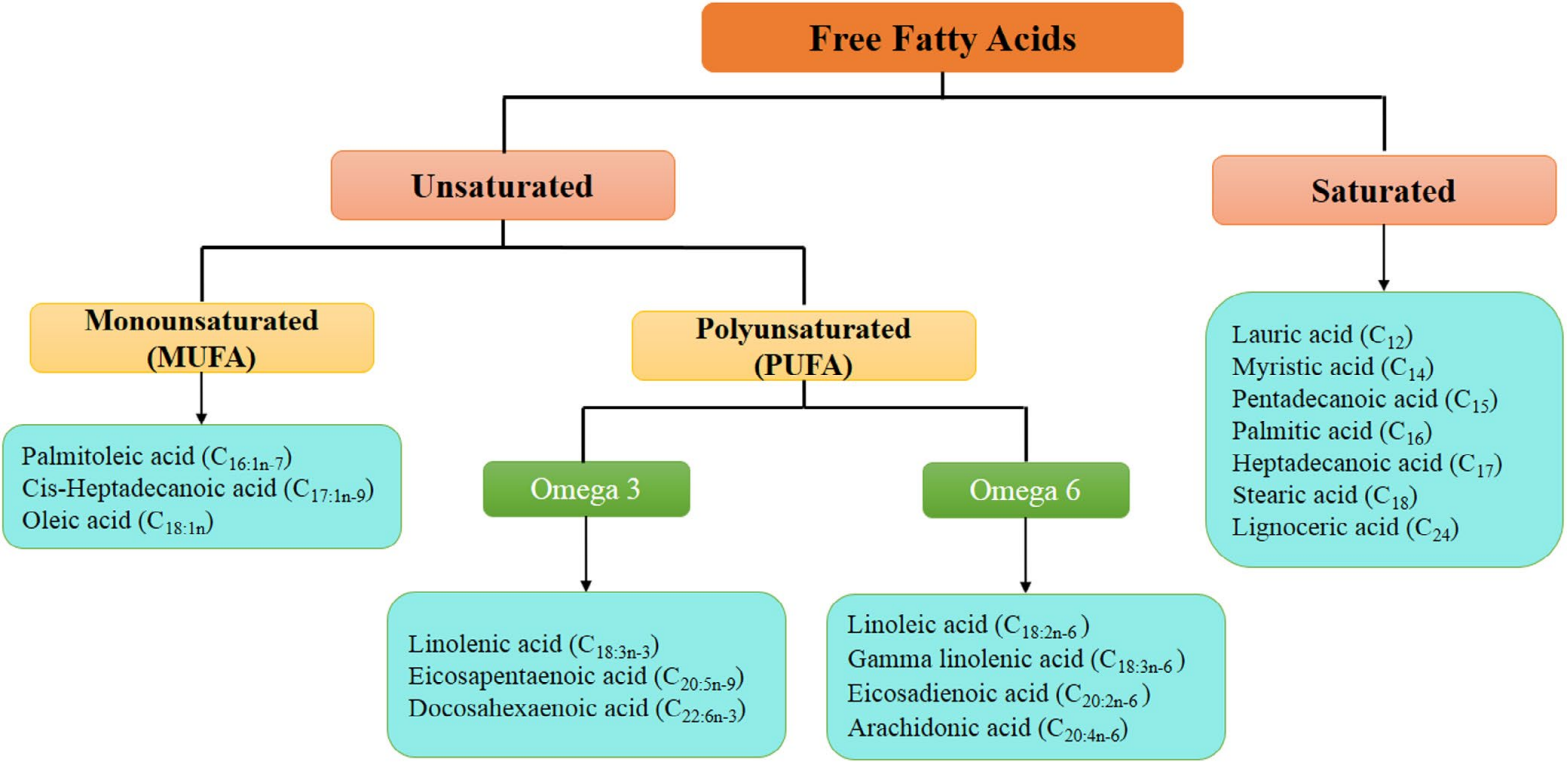
Classification of plasma free fatty acids.

Plasma FFAs are classified by their chemical structure, specifically carbon chain length and double bond position. FFAs are further categorised as saturated fatty acids (SFAs), monounsaturated fatty acids (MUFAs) and polyunsaturated fatty acids (PUFAs), including omega‐3 and omega‐6 families. These subclasses perform distinct physiological functions and may regulate gene expression and metabolic pathways differently (Figure 1). Additionally, FAs, carboxylic acids with aliphatic chains, are classified by chain length: short‐chain FAs (SCFAs; < 6 carbons), medium‐chain FAs (MCFAs; 6–12 carbons), long‐chain FAs (LCFAs; 13–21 carbons) and very long‐chain FAs (VLCFAs; ≥ 22 carbons). SCFAs and MCFAs are quickly oxidised and not stored as triacylglycerols (TAGs) in humans and animals. In contrast, LCFAs, especially those with ≥ 16 carbons, are mainly stored in adipose tissue via triglyceride esterification [12]. Under normal physiological conditions, TAGs are hydrolysed via lipolysis to release free FAs (FFAs), which serve as an energy source and help regulate blood sugar levels during physical activity [12]. However, chronically elevated plasma FFA levels, often observed in severe obesity, indicate metabolic dysregulation and are linked to adverse health outcomes [12, 13]. The concentration of FAs within serum cholesteryl esters (CEs) is typically represented as a percentage of total FAs, highlighting the interconnected nature of these compounds. Changes in the proportion of one FA can impact the levels of others [14]. To address this complexity, studies have employed principal component analysis (PCA) to identify key FA factors within serum, which serve as markers of fat quality, endogenous FA metabolism or a combination of both [15, 16]. This approach provides a clearer view of fat quality factors, offering a more focused analysis than examining the full FA composition in serum lipid esters.

While animal studies have demonstrated that dietary FAs can influence PPAR gene expression, the nature of FAs that can regulate PPARγ gene transcription is unclear [17, 18]. Additionally, no studies have investigated how plasma lipid levels and patterns affect PPARγ gene expression in human adipose tissue. To address this gap, our study aimed to explore the relationship between plasma FAs, both individually and as FA factors, and PPARγ gene expression in VAT and SAT adipose tissues of obese and nonobese adults.

## 2 Material and Methods

### 2.1 Participants

This cross‐sectional study included adults admitted for elective abdominal surgeries at Mostafa Khomeini and Khatam Al‐Anbia hospitals in Tehran, Iran, who met the study criteria. The participants were individuals aged 19 years and older who underwent bariatric surgery or other common abdominal procedures, such as gallbladder surgery or appendectomy. Those excluded from the study were patients with chronic illnesses like diabetes, individuals on fat‐reducing, antiobesity or blood sugar‐regulating medications, those who had followed a specific diet in the past 3 months, patients with sensory impairments or reduced consciousness and individuals hospitalised for more than 3 days prior to the study. Ultimately, 176 adults aged between 19 and 65 were selected for participation. All individuals provided written informed consent. The study adhered to the Declaration of Helsinki and RIES organisational guidelines and received approval from the Ethics Committee of the Research Institute of Endocrine Sciences (RIES) at Shahid Beheshti University of Medical Sciences (NO: IR.SBMU.ENDOCRINE.REC.1400.072).

### 2.2 Blood and Adipose Tissue Sample Collection

Blood samples were taken after 12–14 h of overnight fasting for biochemical analysis in a standard sitting position. Venous blood was drawn from the antecubital vein and centrifuged for 10 min at 1008 × g to separate the serum and plasma. Additionally, the attending surgeon obtained VAT and SAT samples weighing approximately 50–100 mg and placed them in RNase and DNase‐free microtubes. These samples were immediately frozen in liquid nitrogen and stored at −80°C for subsequent RNA extraction to determine PPARγ expression levels.

### 2.3 Anthropometric and Demographic Measurements

Demographic characteristics such as sex, age, history of drug use and medical history were collected and documented. Prior to the surgery, anthropometric measurements were obtained following established protocols [19]. Height was recorded using a wall‐mounted meter accurate to 0.1 cm, and body weight was measured on a Seca scale with an accuracy of 0.1 kg. Abdominal circumference was taken at the level of the umbilicus, situated between the lower ribs and the upper edge of the iliac crest, utilising a tape measure with a precision of 0.5 cm. Body mass index (BMI) was computed in kg/m^2^. Following a 15‐min rest, systolic and diastolic blood pressures (SBP and DBP) were measured twice at 1‐minute intervals with a mercury sphygmomanometer, and the average values were documented [20].

### 2.4 Biochemical Measurements

Fasting plasma glucose (FPG), triglyceride (TG) and total cholesterol (TC) levels were measured using commercial kits (Pars Azmoon Inc., Tehran, Iran). Insulin levels were quantified through an enzyme‐linked immunosorbent assay (ELISA) with the Mercodia kit (Uppsala, Sweden). Inter‐ and intraassay coefficients of variation (CV) were 1.0% for FPG, 0.4% and 2.1% for TG, 0.5% and 1.7% for TC and 1.7% and 2.3% for insulin, respectively.

### 2.5 Physical Activity Assessment

Physical activity levels were evaluated using the Persian version of the long‐form International Physical Activity Questionnaire (IPAQ), developed by the World Health Organisation (WHO). Metabolic equivalent (MET) minutes per week were calculated by multiplying the time spent on each activity by the corresponding MET values for each activity category. The reliability and validity of this questionnaire had been previously verified [21]. Participants were grouped based on their MET scores into three groups: ‘low activity’ (MET < 600 min/week), ‘moderate activity’ (600 < MET < 3000 min/week) and ‘vigorous activity’ (MET > 3000 min/week) [22].

Dietary intake was evaluated by a trained nutritionist using a semi‐quantitative food frequency questionnaire (FFQ), with validated reliability [23, 24]. Participants reported their food consumption patterns over the past year in terms of daily, weekly or monthly intake. Nutrient and energy content were calculated using the United States Department of Agriculture (USDA) Food Composition Table (FCT) [25], while the Iranian FCT was used for local food items [26].

### 2.6 Plasma Fatty Acids Assessment Using Gas Chromatography Flame Ionisation Detector (GC/FID)

As previously described, the plasma FA profile was analysed through the GC/FID method [20]. Sample preparation involved mixing plasma with a 2:1 ratio of chloroform and methanol, followed by adding a 0.9% saline solution. The mixture was then centrifuged at 4500 × g for 10 min at 4°C, allowing the clear chloroform layer containing the FAs to be extracted. The isolated lipids were then dissolved in n‐heptane, and following solvent evaporation using a rotary evaporator, 2 mM potassium hydroxide in methanol was added to the samples. This solution was incubated in a water bath at 70°C for 15 min. Postincubation, the samples were centrifuged at 3500 × g for 5 min at 4°C, and the upper layer containing methyl esters was collected for analysis via flame ionisation detection. Gas chromatography analysis was carried out using a Varian 450 system (City, USA), equipped with a CP‐Sil 88 cyanopropyl siloxane‐coated capillary column (100 m length, 0.25 mm internal diameter and 0.2 μm film thickness) composed of silicon‐based polymers, polyethylene glycols and solid adsorbents. Nitrogen was used as the carrier gas. Methyl esters were identified qualitatively and quantitatively by comparing the chromatograms with a standard FAME Mixture (C4–C24) and calculating the respective peak areas.

### 2.7 Real‐Time Quantitative Reverse Transcription Polymerase Chain Reaction Analysis RT‐qPCR

Tissue samples were weighted and incised; 30–50 mg of VAT and SAT were added to 1 mL TRIzol reagent (Invitrogen, USA) for total RNA extraction, following the manufacturer's instructions. During the extraction, proteins, lipids, carbohydrates and cell debris were removed from the aqueous phase. The quality of the RNA was assessed using a Nanodrop spectrophotometer (ND‐1000, Thermo Scientific, USA) by measuring the 260/280 and 260/230 nm absorption ratios. Additionally, the mRNA integrity was confirmed via gel electrophoresis. DNase I treatment was applied to eliminate DNA contamination and enhance sample purity. Complementary DNA (cDNA) synthesis from the total RNA was performed according to the protocol of a BIOFACT cDNA synthesis kit (BIOFACT, South Korea). Primer sequences used for amplification have been previously reported [27].

RT‐qPCR for PPARγ mRNA was optimised using SYBR Green PCR Master Mix (BIOFACT, Korea) on the Corbett Rotor‐Gene 6000 system (Sydney, Australia), serving as an internal control to normalise mRNA expression levels. The thermal cycling conditions were as described previously [27]. Melting curve analysis verified amplification specificity. All samples were run in duplicate for inter‐assay control, and nontemplate controls (NTC) were included to confirm the reliability of the assay. The relative PPARγ gene expression level in each sample was calculated based on its threshold cycle (*Ct*), normalised to the *Ct* value of the reference gene (GAPDH) and determined using the comparative 2^−*ΔΔCt*^ method as described by Livak and Schmittgen (2001) [28].

### 2.8 Statistical Analyses

Statistical analyses used SPSS 15.0 (SPSS Inc., Chicago, IL, USA), with *p* < 0.05 considered significant. Histograms and Kolmogorov–Smirnov tests checked the normal distribution of variables. Categorical variables were reported as percentages; continuous variables as mean (SD) or median with interquartile range. Participants were grouped into nonobese (BMI < 30 kg/m^2^) and obese (BMI ≥ 30 kg/m^2^). Biochemical differences between groups were evaluated via independent samples t‐test for normally distributed variables, Mann–Whitney *U* test for nonnormally distributed variables and Chi‐square test for qualitative ones. Plasma FA factors were derived using varimax rotation based on 18 types of plasma FAs: lauric, myristic, pentadecanoic, palmitic, palmitoleic, heptadecanoic, cis‐heptadecanoic, stearic, oleic, linoleic, gamma‐linolenic, linolenic, eicosadienoic, eicosatrienoic, arachidonic, eicosapentaenoic (EPA), docosahexaenoic (DHA) and lignoceric acid. Bartlett test of sphericity (*p* < 0.001) and Kaiser–Mayer–Olkin test (0.77) were used to test correlations and sample size adequacy. Three factors were extracted based on eigenvalues > 2, calculated separately for both groups by summing plasma FA frequencies multiplied by factor loadings. The association of individual and extracted plasma FA factors with PPARγ gene expression was assessed using various linear regression models, reporting unstandardised beta (*β*) and 95% confidence intervals (CIs) in crude, age and sex‐adjusted models, with the final model also adjusting for physical activity, serum insulin and energy intake.

## 3 Results

### 3.1 Population Characteristics

Table 1 displays participants' general characteristics and dietary intake data, grouped by nonobese and obese classifications. Participants in the obese group showed significantly higher BMI, FPG, insulin, TG, TC, energy intake, fat intake (as a percentage of energy), plasma SFA levels and higher omega‐3 to omega‐6 FA ratio compared to nonobese subjects. However, obese individuals were younger and had lower levels of physical activity, and consumed a lower percentage of energy from carbohydrates than their nonobese counterparts.

**TABLE 1:** Characteristics of study population among obese and nonobese groups.

Variables	Total (*n* = 167)	BMI < 30 (*n* = 64)	BMI ≥ 30 (*n* = 103)	*p* ^a^
Age (year)	41.4 ± 13.6	48.3 ± 14.9	37.1 ± 10.7	< 0.001
Male (%)	39 (23.4)	18 (28.1)	21 (20.4)	0.168
Female (%)	128 (76.6)	46 (71.9)	82 (79.6)	
BMI (kg/m^2^)	37.9 (25.9, 44.9)	24.6 (22.5, 22.1)	43.9 (39.5, 46.6)	< 0.001
Insulin (mU/mL)	8.67 (4.34, 19.5)	4.51 (2.73, 8.56)	12.5 (6.73, 23.0)	< 0.001
TG (mg/dL)	100 (68.0, 149)	79 (65, 127.7)	109 (72.0, 155)	< 0.003
TC (mg/dL)	179 (152, 208)	167.5 (139.2, 202.7)	182 (161, 209)	0.031
FPG (mg/dL)	89.0 (77.0, 96.7)	84.7 (74.3, 93.0)	90 (79.8, 98.0)	< 0.021
PA (MET‐min/week)	1002 (360, 1793)	1278 (540, 3348)	768 (319, 1644)	< 0.001
Dietary intakes				
Energy (Kcal/d)	2887.2 ± 1000.9	2424.5 ± 747.5	3231 ± 1031.6	< 0.001
Fat (% of energy)	27.5 ± 6.6	25.8 ± 6.02	28.7 ± 6.75	0.001
Carbohydrate (% of energy)	57.2 ± 7.0	59.0 ± 6.64	56.0 ± 6.9	0.001
Protein (% of energy)	14.2 ± 2.28	14.2 ± 1.90	14.2 ± 2.53	0.996
Plasma free fatty acids				
TFAs (mg/mL)	8.83 ± 6.53	8.07 ± 7.47	9.34 ± 5.80	0.230
SFA (mg/mL)	2.21 ± 1.47	1.90 ± 1.27	2.42 ± 1.56	0.022
MUFA (mg/mL)	1.30 (0.83, 2.18)	1.09 (0.83, 1.88)	1.44 (0.82, 2.51)	0.961
PUFA (mg/mL)	3.67 (2.70, 5.61)	3.33 (2.66, 4.54)	4.04 (2.72, 6.05)	0.239
Omega‐3 FA (mg/mL)	0.70 ± 0.33	0.58 ± 0.27	0.77 ± 0.34	< 0.001
Omega‐6 FA (mg/mL)	3.08 (2.06, 4.65)	2.72 (2.06, 3.80)	3.31 (2.07, 5.01)	0.372
Omega‐3:omega‐6 ratio	0.20 (0.14, 0.28)	0.20 (0.14, 0.27)	0.21 (0.13, 0.28)	0.048
SFA (% of TFAs)	26.8 ± 8.98	26.3 ± 9.03	27.1 ± 8.98	0.570
MUFA (% of TFAs)	20.4 ± 7.43	21.2 ± 7.22	19.9 ± 7.55	0.245
PUFA (% of TFAs)	52.8 ± 10.9	52.5 ± 9.37	53.0 ± 11.84	0.741

*Note:* Data represented as mean ± SD or median (25–75 interquartile range) for continuous variables and per cent (%) for categorical variables.

Abbreviations: BMI, body mass index; FPG, fasting plasma glucose; MUFA, monounsaturated fatty acids; PA, physical activity; PUFA, polyunsaturated fatty acids; SAT, subcutaneous adipose tissue; SFA, saturated fatty acids; TC, total cholesterol; TFA, total fatty acids; TG, triglyceride; VAT, visceral adipose tissue.

^a^ Independent t‐test and Mann‐Whitney *U* for quantitative variables and Chi‐square test for qualitative variables.

### 3.2 Gene Expression

**FIGURE 2:**
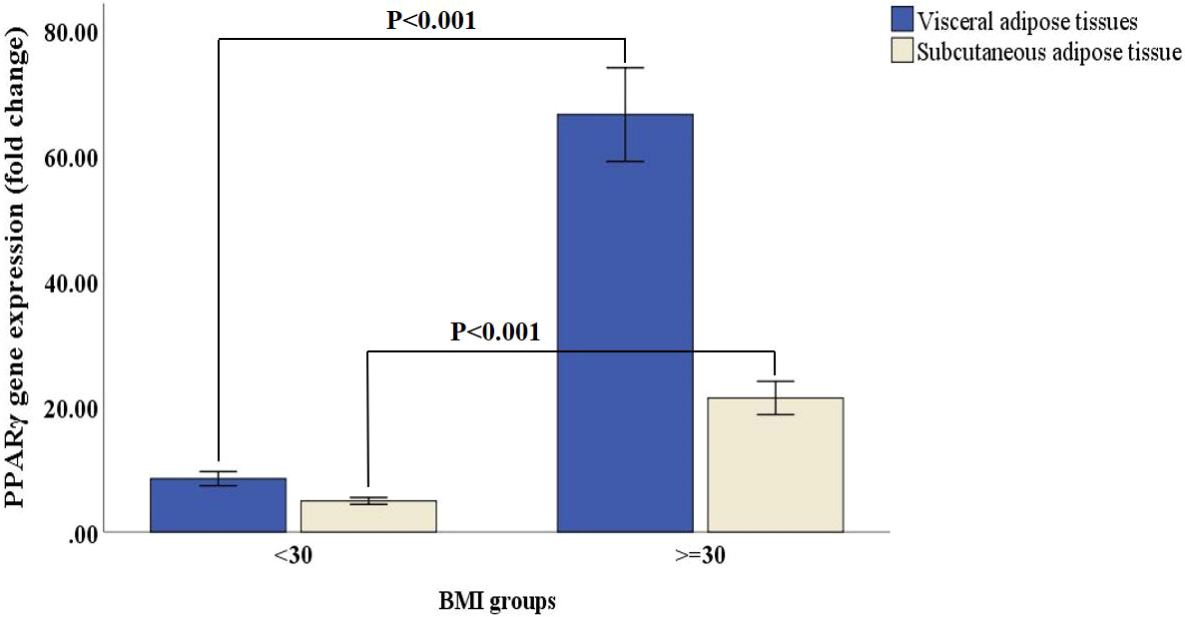
Comparison of relative gene expression of PPARγ in visceral adipose tissues (VAT) and subcutaneous adipose tissue (SAT) of obese (BMI ≥ 30 Kg/m^2^) and nonobese (BMI < 30 Kg/m^2^) subjects. Error bars were defined as 1 standard error of the mean. The displayed *p* values were obtained from the Mann–Whitney *U* test to compare the differences between the two groups. Each sample was analysed in duplicate reactions. The relative gene expression levels were determined using the comparative 2^−*ΔΔCt*^ method.

The median value of VAT and SAT PPARγ gene expression was higher in obese subjects compared to nonobese groups (83.2 vs. 2.4 and 20.4 vs. 3.9, respectively; *p* < 0.001). Moreover, VAT PPARγ mRNA level compared to SAT was elevated in obese individuals (83.2 vs. 20.4; *p* < 0.001), while this was lower in nonobese subjects (2.4 vs. 3.9; *p* = 0.020) (Figure 2).

### 3.3 Plasma Fatty Acids in Obese and Nonobese Subjects

A total of eighteen FFAs were detected using the GC method, which had excellent linearity, repeatability and bias percentages falling within the acceptable limits. The association between plasma FFA levels and obesity status is outlined in Table 2. These results showed that among the 18 known FFAs, the plasma levels of FAs included myristic, pentadecanoic, palmitic, palmitoleic, cis‐heptadecanoic, stearic, gamma‐linolenic, linolenic, eicosatrienoic, arachidonic and EPA acids were higher in obese subjects than in nonobese.

**TABLE 2:** Plasma free fatty acids, among total obese and nonobese participants.

Variables	Carbons	Total (*n* = 167)	BMI < 30 (*n* = 64)	BMI ≥ 30 (*n* = 103)	*p* ^a^
Lauric acid (mg/mL)	12:0^b^	0.10 (0.00, 0.12)	0.11 (0.00, 0.12)	0.09 (0.00, 0.12)	0.891
Myristic acid (mg/mL)	14:0	0.24 (0.20, 0.32)	0.24 (0.19, 0.25)	0.26 (0.21, 0.40)	0.044
Pentadecanoic acid (mg/mL)	15:0	0.00 (0.00, 0.14)	0.00 (0.00, 0.13)	0.13 (0.00, 0.19)	0.006
Palmitic acid (mg/mL)	16:0	0.76 (0.56, 1.05)	0.67 (0.50, 0.88)	0.84 (0.60, 1.14)	0.047
Palmitoleic acid (mg/mL)	16:1n‐7^c^	0.25 (0.18, 0.39)	0.21 (0.14, 0.32)	0.27 (0.20, 0.41)	0.039
Heptadecanoic acid (mg/mL)	17:0	0.15 (0.00, 0.22)	0.15 (0.00, 0.17)	0.16 (0.00, 0.28)	0.208
Cis_Heptadecanoic acid (mg/mL)	17:1n‐9	0.00 (0.00, 0.00)	0.00 (0.00, 0.00)	0.00 (0.00, 0.16)	0.041
Stearic acid (mg/mL)	18:0	0.45 (0.34, 0.59)	0.40 (0.30, 0.52)	0.50 (0.36, 0.65)	0.027
Oleic acid (mg/mL)	18:1	0.95 (0.54, 1.50)	0.85 (0.60, 1.36)	1.06 (0.52, 1.63)	0.500
Linoleic acid (mg/mL)	18:2n‐6	2.50 (1.60, 4.0)	2.25 (1.60, 3.23)	2.72 (1.54, 4.30)	0.569
Gamma‐linolenic acid (mg/mL)	18:3n‐6	0.15 (0.60, 0.18)	0.14 (0.00, 0.17)	0.15 (0.10, 0.19)	0.008
Linolenic acid (mg/mL)	18:3n‐3	0.16 (0.09, 0.19)	0.15 (0.02, 0.17)	0.17 (0.13, 0.21)	0.011
Eicosadienoic acid (mg/mL)	20:2n‐6	0.00 (0.00, 0.3)	0.00 (0.00, 0.00)	0.00 (0.00, 0.04)	0.055
Eicosatrienoic acid (mg/mL)	20:3n‐9	0.25 (0.20, 0.33)	0.22 (0.17, 0.28)	0.28 (0.22, 0.40)	< 0.001
Arachidonic acid (mg/mL)	20:4n‐6	0.40 (0.27, 0.56)	0.34 (0.21, 0.44)	0.46 (0.32, 0.65)	< 0.001
Eicosapentaenoic acid (mg/mL)	20:5n‐9	0.00 (0.00, 0.14)	0.00 (0.00, 0.14)	0.05 (0.00, 0.15)	0.009
Docosahexaenoic acid (mg/mL)	22:6n‐3	0.20 (0.16, 0.25)	0.19 (0.14, 0.22)	0.20 (0.16, 0.26)	0.115
Lignoceric acid (mg/mL)	24:0	0.04 (0.00, 0.12)	0.02 (0.00, 0.12)	0.05 (0.00, 0.13)	0.833

*Note:* Data represented as median (25–75 interquartile range).

^a^ Mann–Whitney U test for quantitative variables and Chi‐square test for qualitative variables.

^b^ Number of carbons.

^c^ Number of unsaturated bonds of carbon.

### 3.4 Factor Analysis of Individual FAs

PCA analysis was used, and three new plasma FA factors in every group were identified (factors I, II and III) (Table 3). In nonobese and obese groups, the first three factors explained 69% and 71% of the total variance, respectively. In the nonobese group, factor I (eigenvalue = 6.42) was identified by higher factor loading (0.60–0.92) for the relative amount of medium and long chain FAs including lauric (C_12_), pentadecanoic (C_15_), arachidonic (C_20:4n‐6_), DHA (C_22:6n‐3_) and lignoceric acids (C_24_). We characterised factor II (eigenvalue = 3.67) with high factor loading (0.81–0.95) for palmitic (C_16_), stearic (C_18_), oleic (C_18:1n_) and linoleic acids (C_18:2n_). Factor III (eigenvalue = 2.30) was characterised by high factor loading (0.5–0.95) for myristic (C_14_), heptadecanoic (C_17_), cis‐heptadecanoic (C_17:1n‐9_) and EPA acids (C_20:5n‐9_). In the obese group, factor I (eigenvalue = 7.47) characterised by high loads (ranges between 0.62 and 0.79) of lauric (C_12_), palmitic (C_16_), palmitoleic (C_16:1n‐7_), gamma‐linolenic (C_18:3n‐6_), linolenic (C_18:3n‐3_) and eicosatrienoic acids (C_20:3n‐9_). Factor II (eigenvalue = 3.30) was ascertained by high loading factor (0.78–0.98) of myristic (C_14_), pentadecanoic (C_15_), heptadecanoic (C_17_) and cis‐heptadecanoic acids (C_17:1n‐9_) and factor III (eigenvalue = 2.00) was rich in stearic (C_18_), oleic (C_18:1n_) and linoleic acids (C_18:2n‐6_).

**TABLE 3:** Factor loading matrix for major plasma free fatty acids factors identified by factor analysis among the obese and nonobese participants.

Plasma fatty acids	BMI < 30 (*n* = 64)			BMI ≥ 30 (*n* = 103)		
	Fatty acid factors*			Fatty acid factors		
	Factor I	Factor II	Factor III	Factor I	Factor II	Factor III
Lauric acid C_12:0_	0.815			0.625		
Myristic acid C_14:0_			0.847		0.956	
Pentadecanoic acid C_15:0_	0.748		0.557	0.325	0.785	
Palmitic acid C_16:0_		0.817		0.658		0.642
Palmitoleic acid C_16:1n‐7_	0.368	0.348		0.767		
Heptadecanoic acid C_17:0_	0.606		0.699		0.968	
Cis_Heptadecanoic acid C_17:1n‐9_			0.950		0.980	
Stearic acid C_18:0_	0.303	0.878		0.373		0.803
Oleic acid C_18:1_		0.959				0.928
Linoleic acid C_18:2n‐6_		0.943				0.964
Gamma‐Linolenic acid C_18:3n‐6_				0.660		0.364
Linolenic acid C_18:3n‐3_		0.512		0.749		
Eicosadienoic acid C_20:2n‐6_				0.460		
Eicosatrienoic acid C_20:3n‐9_				0.793		
Arachidonic acid C_20:4n‐6_	0.904			0.477		0.307
Eicosapentaenoic acid C_20:5n‐9_	0.313	0.386	0.506			
Docosahexaenoic acid C_22:6n‐3_	0.924			0.558		
Lignoceric acid C_24:0_	0.785					
Cumulative variance explained (%)*	35.7	56.1	69.0	41.5	59.9	71.0

* Percentage of variance in total plasma fatty acids is explained by factors (refer to Cumulative Variance Explained (%)).

### 3.5 Association of Plasma FAs Subgroup and PPARγ Gene Expression

Linear regression analysis assessed the association between plasma FAs subgroups and PPARγ gene expression in VAT and SAT. The relationship between PPAR γ gene expression and plasma FAs subgroups among obese and nonobese groups is summarised in Table 4. These results showed that after controlling for confounding factors such as age, sex, physical activity, serum insulin and energy intake, PPARγ expression in SAT was negatively associated with SFA and SFA% and positively associated with PUFA% among nonobese individuals. Furthermore, VAT PPARγ expression had a significant negative association with TFA, SFA, MUFA, PUFA and omega‐6 FA among obese individuals.

**TABLE 4:** Association of plasma free fatty acid subgroups with PPARγ gene expression in visceral and subcutaneous adipose tissue.

	BMI < 30 (*n* = 64)				BMI ≥ 30 (*n* = 103)			
	Visceral		Subcutaneous		Visceral		Subcutaneous	
	Beta (95% CI)	*p*	Beta (95% CI)	*p*	Beta (95% CI)	*p*	Beta (95% CI)	*p*
Total fatty acids								
Crude	−0.00 (−0.02, 0.02)	0.905	−0.00 (−0.02, 0.01)	0.772	−0.01 (−0.03, 0.00)	0.050	0.00 (−0.01, 0.03)	0.586
Model 1^a^	−0.00 (−0.02, 0.02)	0.895	−0.00 (−0.02, 0.01)	0.787	−0.02 (−0.04, −0.00)	0.030	0.00 (−0.01, 0.03)	0.504
Model 2^b^	−0.00 (−0.02, 0.00)	0.221	−0.00 (−0.02, 0.01)	0.446	−0.02 (−0.04, −0.00)	0.017	0.00 (−0.01, 0.03)	0.493
SFA								
Crude	−0.06 (−0.18, 0.05)	0.263	−0.12 (−0.22, −0.03)	0.009	−0.07 (−0.14, −0.00)	0.040	−0.02 (−0.11, 0.06)	0.640
Model 1	−0.06 (−0.18, 0.05)	0.312	−0.12 (−0.22, −0.03)	0.013	−0.07 (−0.14, −0.00)	0.036	−0.02 (−0.11, 0.06)	0.631
Model 2	−0.05 (−0.13, 0.02)	0.162	−0.12 (−0.21, −0.02)	0.019	−0.06 (−0.13, −0.00)	0.048	−0.02 (−0.11, 0.07)	0.703
MUFA								
Crude	−0.01 (−0.07, 0.04)	0.596	0.00 (−0.05, 0.05)	0.979	−0.07 (−0.14, −0.00)	0.045	0.01 (−0.07, 0.10)	0.739
Model 1	−0.02 (−0.08, 0.04)	0.553	0.00 (−0.05, 0.05)	0.992	−0.08 (−0.16, −0.01)	0.021	0.02 (−0.07, 0.12)	0.600
Model 2	−0.03 (−0.07, 0.01)	0.207	−0.01 (−0.06, 0.04)	0.657	−0.08 (−0.15, −0.01)	0.020	0.02 (−0.07, 0.12)	0.608
PUFA								
Crude	0.00 (−0.02, 0.04)	0.681	0.00 (−0.02, 0.03)	0.816	−0.02 (−0.05, 0.00)	0.140	0.02 (−0.02, 0.06)	0.333
Model 1	0.00 (−0.02, 0.04)	0.695	0.00 (−0.02, 0.03)	0.809	−0.02 (−0.06, 0.00)	0.098	0.02 (−0.02, 0.06)	0.271
Model 2	−0.01 (−0.03, 0.01)	0.320	−0.00 (−0.03, 0.02)	0.698	−0.03 (−0.06, −0.00)	0.039	0.02 (−0.02, 0.06)	0.275
Omega‐3 fatty acids								
Crude	−0.32 (−0.90, 0.24)	0.254	−0.32 (−0.89, 0.24)	0.254	−0.09 (−0.40, 0.22)	0.577	−0.09 (−0.40, 0.22)	0.577
Model 1	−0.30 (−0.89, 0.27)	0.298	−0.30 (−0.89, 0.27)	0.298	−0.06 (−0.38, 0.25)	0.698	−0.06 (−0.38, 0.25)	0.689
Model 2	−0.23 (−0.62, 0.16)	0.241	−0.23 (−0.62, 0.15)	0.241	−0.15 (−0.45, 0.15)	0.331	−0.15 (−0.45, 0.15)	0.331
Omega‐6 fatty acids								
Crude	0.00 (−0.02, 0.04)	0.626	0.00 (−0.02, 0.03)	0.721	−0.02 (−0.05, 0.00)	0.137	0.02 (−0.02, 0.06)	0.301
Model 1	0.00 (−0.02, 0.04)	0.645	0.00 (−0.02, 0.03)	0.720	−0.02 (−0.06, 0.00)	0.091	0.02 (−0.01, 0.07)	0.231
Model 2	−0.01 (−0.03, 0.01)	0.347	−0.00 (−0.03, 0.02)	0.777	−0.03 (−0.06, −0.00)	0.040	0.02 (−0.01, 0.07)	0.242
Omega‐3:omega‐6 ratio								
Crude	0.03 (−1.10, 1.16)	0.959	−0.56 (−1.50, 0.38)	0.237	0.17 (−0.16, 0.52)	0.310	−0.08 (−0.52, 0.35)	0.700
Model 1	0.00 (−1.17, 1.18)	0.997	−0.59 (−1.57, 0.39)	0.234	0.18 (−0.15, 0.52)	0.285	−0.09 (−0.53, 0.34)	0.665
Model 2	0.24 (−0.57, 1.06)	0.551	−0.37 (−1.40, 0.66)	0.472	0.15 (−0.17, 0.48)	0.352	−0.07 (−0.51, 0.37)	0.756
SFA (% of total fatty acids)								
Crude	−0.00 (−0.02, 0.01)	0.871	−0.02 (−0.04, −0.01)	< 0.001	0.00 (−0.01, 0.01)	0.977	−0.00 (−0.02, 0.01)	0.448
Model 1	−0.00 (−0.02, 0.01)	0.893	−0.02 (−0.04, −0.01)	< 0.001	0.00 (−0.01, 0.01)	0.881	−0.00 (−0.02, 0.00)	0.375
Model 2	−0.00 (−0.01, 0.01)	0.771	−0.02 (−0.04, −0.01)	0.002	0.00 (−0.00, 0.01)	0.679	−0.00 (−0.02, 0.01)	0.460
MUFA (% of total fatty acids)								
Crude	−0.01 (−0.03, 0.01)	0.277	−0.00 (−0.02, 0.01)	0.661	−0.00 (−0.01, 0.00)	0.480	−0.00 (−0.02, 0.01)	0.567
Model 1	−0.01 (−0.03, 0.00)	0.198	−0.00 (−0.02, 0.01)	0.517	−0.00 (−0.02, 0.00)	0.343	−0.00 (−0.02, 0.01)	0.724
Model 2	−0.00 (−0.01, 0.01)	0.772	−0.00 (−0.02, 0.01)	0.571	−0.00 (−0.01, 0.00)	0.518	−0.00 (−0.02, 0.01)	0.703
PUFA (% of total fatty acids)								
Crude	0.00 (−0.00, 0.02)	0.337	0.02 (0.01, 0.03)	< 0.001	0.00 (−0.00, 0.01)	0.636	0.00 (−0.00, 0.01)	0.372
Model 1	0.00 (−0.00, 0.02)	0.283	0.02 (0.01, 0.03)	< 0.001	0.00 (−0.00, 0.01)	0.627	0.00 (−0.00, 0.01)	0.373
Model 2	0.00 (−0.01, 0.01)	0.979	0.02 (0.008, 0.03)	0.002	0.00 (−0.00, 0.00)	0.922	0.00 (−0.00, 0.01)	0.429

^a^ Adjusted for age and sex.

^b^ Adjusted for Model 1 and physical activity, serum insulin and energy intake.

### 3.6 Association of Plasma FAs Factors and PPARγ Gene Expression

The results using multivariable linear regression analysis demonstrated an inverse correlation between factor I of FAs and PPARγ expression in the nonobese group (*β* = −0.16; 95% CI: −0.29, −0.04, *p* = 0.008 in SAT) (Table 5). After adjustment for age, sex, physical activity, serum insulin and energy intake, only factor I was associated with PPARγ gene expression in SAT in the nonobese group (*β* = −0.15; 95% CI: −0.27, −0.02, *p* = 0.027). Other factors were not associated with PPARγ gene expression in VAT and SAT.

**TABLE 5:** Association of plasma free fatty acid factors with PPARγ gene expression in visceral and subcutaneous adipose tissue.

	BMI < 30 (*n* = 64)				BMI ≥ 30 (*n* = 103)			
	Visceral		Subcutaneous		Visceral		Subcutaneous	
	Beta (95% CI)	*p*	Beta (95% CI)	*p*	Beta (95% CI)	*p*	Beta (95% CI)	*p*
Factor 1								
Crude	−0.13 (−0.28, 0.02)	0.085	−0.16 (−0.29, −0.04)	0.008	−0.02 (−0.13, 0.08)	0.649	−0.05 (−0.19, 0.08)	0.443
Model 1^a^	−0.12 (−0.28, 0.02)	0.101	−0.16 (−0.29, −0.04)	0.011	−0.00 (−0.12, 0.10)	0.878	−0.08 (−0.21, 0.06)	0.263
Model 2^b^	−0.05 (−0.16, 0.05)	0.319	−0.15 (−0.27, −0.02)	0.027	−0.02 (−0.13, 0.08)	0.661	−0.07 (−0.21, 0.07)	0.342
Factor 2								
Crude	−0.01 (−0.16, 0.14)	0.886	−0.02 (−0.15, 0.10)	0.753	−0.07 (−0.18, 0.04)	0.196	−0.07 (−0.20, 0.07)	0.324
Model 1	−0.01 (−0.17, 0.14)	0.850	−0.02 (−0.15, 0.10)	0.741	−0.08 (−0.19, 0.02)	0.131	−0.05 (−0.19, 0.08)	0.432
Model 2	−0.05 (−0.16, 0.05)	0.342	−0.06 (−0.20, 0.07)	0.352	−0.05 (−0.15, 0.05)	0.307	−0.05 (−0.20, 0.08)	0.442
Factor 3								
Crude	0.02 (−0.13, 0.17)	0.772	−0.07 (−0.19, 0.05)	0.270	−0.07 (−0.18, 0.04)	0.202	0.06 (−0.08, 0.20)	0.397
Model 1	0.03 (−0.12, 0.18)	0.683	−0.06 (−0.19, 0.06)	0.318	−0.09 (−0.20, 0.02)	0.125	0.07 (−0.06, 0.22)	0.293
Model 2	−0.01 (−0.13, 0.10)	0.784	−0.04 (−0.18, 0.10)	0.598	−0.10 (−0.20, 0.00)	0.061	0.07 (−0.07, 0.22)	0.306

^a^ Adjusted for age and sex.

^b^ Adjusted for Model 1 and physical activity, serum insulin and energy intake.

## 4 Discussion

This study is the first to explore the relationship between PPARγ gene expression in SAT and VAT adipose tissues and plasma FFA profiles in nondiabetic individuals across a wide BMI spectrum. We found significantly higher PPARγ expression in both VAT and SAT of obese subjects compared to nonobese groups. Using PCA, we identified three distinct FA factors (I, II and III), which accounted for a substantial proportion of the variance in FA profiles. After adjusting for confounders such as age, gender, physical activity, serum insulin and energy intake, we observed an inverse association between PPARγ expression and specific FA profiles. Notably, in nonobese individuals, SAT PPARγ expression was inversely related to SFAs and factor I (comprising lauric, pentadecanoic, arachidonic, docosahexaenoic and lignoceric acids). In obese individuals, VAT PPARγ expression demonstrated significant inverse associations with total FAs, SFAs, MUFAs, PUFAs and omega‐6 FAs, as well as a marginal inverse relationship with factor III (including stearic, oleic and linoleic acids).

Dysfunction in PPARγ is closely linked with insulin resistance and obesity. However, the relationship between PPARγ expression and plasma FA profiles has not been extensively studied. While earlier investigations have explored the effects of dietary FAs on PPARγ activity in animal models [17, 18], human studies remain scarce. Our results are consistent with prior observations that elevated PPARγ expression in adipose tissue is associated with obesity [29]. However, the inverse associations we observed between PPARγ expression and specific FAs, such as oleic and omega‐6 FAs, provide new insights into the complex interplay between dietary lipids and metabolic regulation.

Here, we found that VAT PPARγ expression was inversely associated with plasma levels of TFA, SFA, MUFA, PUFA and omega‐6 FA in obese individuals. These results align with previous research, which identified a negative correlation between VAT PPARγ expression and dietary intake of linoleic and oleic acids in obese subjects [30]. One possible explanation is that SFAs may directly suppress PPARγ activity, leading to metabolic dysregulation. Excessive activation of PPARγ can lead to fat accumulation and hepatic steatosis, while inhibition of this pathway may have antiobesity and antidiabetic effects [31, 32]. On the other hand, trans and saturated fatty acids not only reduce PPARγ expression but are also associated with increased inflammation and impaired lipid metabolism, which can raise the risk of metabolic diseases such as nonalcoholic fatty liver disease and metabolic syndrome [33, 34]. Therefore, regulating PPARγ expression and the type of fatty acids consumed can play a key role in preventing or progressing obesity‐related metabolic disorders.

Additionally, the marginal inverse association between VAT PPARγ expression and factor III (containing oleic acids) highlights the potential protective role of monounsaturated FAs in metabolic health, as supported by recent studies [35, 36]. Our data showed a negative relationship between VAT PPARγ expression and factor III, which includes oleic acid; however, this association did not reach statistical significance, likely due to the small sample size in the study. Our findings suggest that these FAs may modulate PPARγ activity through mechanisms involving stearoyl‐CoA desaturase 1 (SCD1), an enzyme implicated in lipid metabolism and insulin resistance [37, 38]. Conversely, SCD1 deficiency has been associated with higher levels of PUFA, EPA and DHA, which may stimulate β‐oxidation by activating PPAR [39]. While omega‐3 FAs can activate PPARs, DHA and EPA, two important components, are weaker agonists and exhibit reduced efficacy. Linolenic and arachidonic acids (omega‐3 and omega‐6, respectively) show similar potency to DHA and EPA in PPAR activation [40]. Reflecting our findings, research by Hanna et al. demonstrated that unsaturated fatty acids (UFA) can decrease the nuclear content of SREBP‐1, leading to reduced expression of lipogenic genes, including PPARγ [41].

Moreover, we observed an inverse association between SAT PPARγ expression and SFA levels as well as factor I in the nonobese group, including lauric, pentadecanoic, stearic, lignoceric, arachidonic and DHA acids. Experimental studies indicated that fish and flaxseed oils can enhance PPARα and PPARγ mRNA levels. In contrast to our findings, Rahmani et al. reported a significant increase in PPARγ gene expression following 12 weeks of fish oil supplementation in individuals with PCOS [42]. Other studies have examined the impact of omega‐3 supplementation on fibroblast growth factor 21 (FGF‐21) levels, a metabolic regulator primarily produced by the liver in white adipose tissue [43, 44]. Omega‐3 FAs have been found to reduce circulating FGF‐21 levels and enhance FGF‐21 sensitivity via a PPARγ‐dependent mechanism. A study by Naeini et al. demonstrated that DHA‐enriched fish oil supplements increased PPARγ activity in patients with type 2 diabetes mellitus, particularly in peripheral blood mononuclear cells (PBMCs) [45]. Recent evidence indicates a complex relationship between PPARγ activity, VLCFA metabolism and β‐oxidation, which could significantly affect metabolic health. While PPARγ primarily regulates adipocyte differentiation and lipid storage, it also affects the expression of genes related to fatty acid uptake and oxidation, including those necessary for peroxisomal and mitochondrial β‐oxidation pathways [46, 47]. VLCFAs, including docosahexaenoic acid (DHA, C22:6n‐3) and lignoceric acid (C24:0), need initial catabolism in peroxisomes before further β‐oxidation in mitochondria. Disruption of VLCFA metabolism in peroxisomal disorders causes accumulation linked to insulin resistance and adipose tissue dysfunction. Additionally, studies show that PPARγ activation can upregulate genes for enzymes involved in peroxisomal β‐oxidation, facilitating VLCFA clearance and potentially reducing lipotoxicity [48]. Conversely, impaired PPARγ signalling or dysregulated VLCFA metabolism may contribute to ectopic lipid accumulation, chronic inflammation and the development of obesity‐related metabolic disorders such as obesity and type 2 diabetes [49].

Our findings reveal depot‐specific associations between plasma VLCFAs and PPARγ expression, supporting the notion that regulating VLCFA metabolism and β‐oxidation via PPARγ is crucial for adipose tissue health and metabolic homeostasis. The observed inverse relationship between certain VLCFA factors and PPARγ expression in SAT of nonobese individuals may indicate a compensatory mechanism to boost FA oxidation and prevent lipotoxicity. These insights highlight PPARγ's important role linking FA metabolism, peroxisomal and mitochondrial β‐oxidation and the development or prevention of metabolic disease. Further research is needed to clarify the molecular mechanisms of these interactions and explore their therapeutic potential in obesity and related metabolic disorders.

The differences in plasma FA profiles between individuals with BMI ≥ 30 (obese) and those with BMI < 30 (nonobese) reflect metabolic inflection points linked to obesity‐related dysregulation. Studies show obese individuals have altered plasma lipid compositions, with increased triglycerides and metabolites like 18‐hydroxycortisol, and decreased phospholipids such as phosphatidylcholine and lysophosphatidylcholine, correlating strongly with measures of adiposity [50, 51]. These changes indicate a shift in lipid metabolism that occurs during the transition from being overweight to obesity.

Moreover, bariatric surgery studies reveal that severe obesity is characterised by elevated plasma FFAs, including saturated and unsaturated species such as palmitate, oleate and linoleate, which decrease significantly after surgical weight loss, indicating improved lipid metabolism and glycaemic control [52]. This supports the notion that BMI thresholds around 30 kg/m^2^ may mark a metabolic tipping point where lipid handling and FA turnover become markedly impaired.

Mechanistically, elevated FFAs in obese individuals alter key metabolic regulators like PPARγ in adipose tissue, affecting adipocyte differentiation and lipid storage. Obese adults have higher PPARγ expression in visceral and subcutaneous fat, linked to altering saturated, monounsaturated and polyunsaturated FA profiles, indicating adaptations in lipid signalling that may worsen metabolic dysfunction. Furthermore, obesity is associated with increased basal lipolysis and impaired insulin‐mediated FFA suppression, especially in upper body fat, contributing to systemic metabolic disturbances [53].

These findings indicate that plasma FA profile differences across BMI categories reflect qualitative shifts in lipid metabolism. Such shifts likely signify points where compensatory mechanisms fail, resulting in adipose tissue dysfunction, insulin resistance and increased cardiometabolic risk. Understanding these lipid profile changes provides insight into obesity pathophysiology and potential therapeutic targets for improving metabolism in individuals with a BMI ≥ 30 [54].

PPARγ, a key regulator of lipid metabolism, cell proliferation and inflammation [55], plays a crucial role in adipocyte differentiation [56]. Fatty acids can influence these biological processes through signalling pathways that activate or suppress DNA transcription [57], although the precise mechanisms remain unclear. Previous studies have shown that FAs composition can contribute to the development of insulin resistance, type 2 diabetes [58] and metabolic syndrome [15].

Despite its innovative contributions, our study presents several limitations. First, the cross‐sectional design restricts our ability to establish causality between plasma FA profiles and PPARγ mRNA levels. Longitudinal studies are essential for evaluating how changes in FA profiles over time affect PPARγ activity and metabolic outcomes. Second, the study population, which consists of individuals undergoing elective surgeries, may restrict the generalisability of our findings. Future research should involve broader, more diverse cohorts to improve external validity. Third, while our lipidomics approach offers a comprehensive view of FA profiles, the underlying molecular mechanisms that link specific FAs to PPARγ expression remain unclear. Finally, due to limited resources, we were unable to assess the activity of key downstream enzymes involved in peroxisomal and mitochondrial β‐oxidation pathways, such as CPT1, ACOX1 and MCAD, which are transcriptionally regulated by PPARγ. Future studies should address this gap to better understand the underlying mechanisms.

## 5 Conclusion

Our study highlights the intricate association between plasma FA profiles and PPARγ gene expression in adipose tissue, particularly in the context of obesity. The inverse associations observed between PPARγ expression and specific FAs, such as oleic and omega‐6 FAs, suggest that these lipids may influence metabolic pathways involved in lipid storage, insulin sensitivity and inflammation. Additionally, the impact of omega‐3 FAs on PPARγ activity further highlights the complexity of dietary lipid interactions in obesity and metabolic disorders. Author contributions: MS, MZ and RA designed and drafted the manuscript, collected the references, and carried out the primary literature search. FT, MA, GA, EY, MH and AKH modify the manuscript and participated in discussions. All authors read and approved the final manuscript.

## Author Contributions

M.S., M.Z. and R.A. designed and drafted the manuscript, collected the references, and carried out the primary literature search. F.T., M.A., G.A., E.Y., M.H. and A.K. modify the manuscript and participated in discussions. All authors read and approved the final manuscript.

## Conflicts of Interest

The authors declare no conflicts of interest.

## Data Availability

The data that support the findings of this study are available from the corresponding author upon reasonable request.
